# Storage temperature dictates the ability of chicken embryos to successfully resume development by regulating expression of blastulation and gastrulation genes

**DOI:** 10.3389/fphys.2022.960061

**Published:** 2022-12-16

**Authors:** Narayan Pokhrel, Olga Genin, Dalit Sela-Donenfeld, Yuval Cinnamon

**Affiliations:** ^1^ Agriculture Research Organization, Volcani Center, Department of Poultry and Aquaculture Science, Rishon LeTsiyon, Israel; ^2^ Koret School of Veterinary Medicine, The Robert H. Smith Faculty of Agriculture, Food and Environment, The Hebrew University of Jerusalem, Rehovot, Israel

**Keywords:** Egg storage conditions, Embryonic survival, Resumption phase, Prolong egg storage, Brachyury (TBX), Nanog, ID2

## Abstract

The avian embryo has a remarkable ability that allows it to suspend its development during blastulation for a long time at low temperatures, and to resume normal development when incubated. This ability is used by poultry hatcheries to store eggs prior to incubation. We have previously found that this ability correlates with the temperature during storage; embryos recover much better following prolonged storage at 12°C rather than at 18°C. However, the molecular and cellular mechanisms underlying these differences are poorly understood. To successfully resume development following storage, the embryo has to shift from the blastulation phase to gastrulation. Several genes are known to partake in the blastulation-to-gastrulation transition under normal conditions, such as the pluripotency-related genes *Inhibitor of DNA Binding 2 (ID2) and NANOG* that are expressed during blastulation, and the gastrulation-regulating genes *NODAL* and *Brachyury (TBXT)*. However, their expression and activity following storage is unknown. To elucidate the molecular mechanisms that initiate the ability to successfully transit from blastulation to gastrulation following storage, embryos were stored for 28 days at 12°C or 18°C, and were assessed either prior to incubation, 12, or 18 h of incubation at 37.8°C. Immediately following storage at 18°C group showed remarkable impaired morphology compared to the blastoderm of the 12°C group and of non-stored control embryos. Concurrently with these, expression of *ID2* and *NANOG* was maintained following storage at 12°C similar to the control group, but was significantly reduced upon storage at 18°C. Nevertheless, when the 18°C-stored embryos were incubated, the morphology and the reduced genes were reverted to resemble those of the 12°C group. At variance, key gastrulation genes, *NODAL* and its downstream effector *Brachyury* (*TBXT*), which were similarly expressed in the control and the 12°C group, were not restored in the 18°C embryos following incubation. Notably, ectopic administration of Activin rescued *NODAL* and *TBXT* expression in the 18°C group, indicating that these embryos maintain the potential to initiate. Collectively, this study suggests a temperature-dependent mechanisms that direct the transition from blastulation to gastrulation. These mechanisms promote a successful developmental resumption following prolonged storage at low temperatures.

## Introduction

Avian embryos undergoing blastulation have a unique ability to arrest their development and remain viable at low temperature for a long time. This ability allows to store eggs prior to incubation (Cai et al., 2019; Pokhrel et al., 2021b). Highly critical for embryonic survival, the storage temperature affects the ability of embryos to successfully resume development and hatch by the end of incubation (Fasenko, 2007). Extending storage duration, without affecting hatchability and chick quality may contribute to poultry hatcheries in planning eggs storage and conditions. Following prolonged time, of up to 28 days, embryos stored at 12°C have better chances to successfully resume development, than at 18°C (Pokhrel et al., 2018; Pokhrel et al., 2021b). Thus, the ability to recover from storage during the first hours of incubation is a manifestation of processes occurring during storage. However, the cellular and molecular mechanisms involved in embryonic recovery following storage are poorly understood.

To successfully resume development, the first steps of embryogenesis at the beginning of incubation are the completion of the blastulation process (Eyal-Giladi and Kochav, 1976; Kochav et al., 1980) and the transitioning to gastrulation, which is manifested by the formation of the Primitive Streak (PS), which occur in stages 2–3 H&H (Hamburger and Hamilton., 1951; Vasiev et al., 2010; Parfitt and Shen, 2014). Relying on cellular remodeling, these events require that the embryonic cytoarchitecture should be preserved during storage. Notably, embryos stored for 28 days at 12°C, maintain their cytoarchitectural structure, similarly to freshly laid embryos, around stage X EG&K (Eyal-Giladi and Kochav, 1976; Pokhrel et al., 2017; Pokhrel et al., 2018). This is facilitated by cell cycle arrest at the G2/M transition, which is regulated by the checkpoint kinase, WEE1 (Pokhrel et al., 2022). In contrast, embryos stored for 28 days at 18°C undergo substantiate cellular remodeling, including overall tissue thickening, formation of deep recesses at the dorsal aspect of the central disc (Area Pellucida, AP), and cells clustering at the ventral side of the AP. These maladaptive changes correlate with poor embryonic survivability and hatchability (Pokhrel et al., 2018).

Several signaling pathways are involved in the blastulation-to-gastrulation transition (Parfitt and Shen, 2014). For example, in blastulating embryos, the Bone Morphogenetic Protein 4 (*BMP4*) signaling pathway is active in the surrounding ring of the embryo, known as the Area Opaca (AO), as well as in the AP, and in cells polyingressing from the epiblast (Streit et al., 1998). In mouse embryonic stem cells, *BMP4* was found to regulate pluripotency by elevating the expression of its downstream effectors—Inhibitor of DNA Binding 2 (*ID2*), and the homeobox transcription factor *NANOG* (Hollnagel et al., 1999; Ying et al., 2003; Qi et al., 2004; Morikawa et al., 2016). Notably, mouse embryonic stem cells and chick blastoderm share similar gene regulatory networks in regulating pluripotency (Parfitt and Shen, 2014; Jean et al., 2015). *Chordin* and *Noggin*, two main BMP4 protein antagonists (Streit et al., 1998), are also expressed during blastulation and gastrulation at the posterior border of the AP (Koller’s sickle region) and at the PS, respectively (Bertocchini and Stern 2008; Vasiev et al., 2010). Hence, *BMP4* activity becomes restricted upon gastrulation initiation (Morgani and Hadjantonakis, 2020).

Concomitant with reduced *BMP4* expression in the onset of gastrulation, *NANOG* expression is also downregulated in the PS (Lavial et al., 2007), whereas, the signaling protein NODAL–which regulates PS formation and mesodermal induction, becomes apparent along the PS (Zimmerman et al., 1996; Bertocchini and Stern, 2002; Umulis et al., 2009; Pereira et al., 2012). Highlighting its evolutionary conserved role in mesoderm induction, *NODAL* is also the earliest marker expressed in presumptive mesoderm in *Xenopus* (Agius et al., 2000). Recently, *NODAL* expression was shown to be induced by Activin, through the same TGFβ receptor subtypes as NODAL protein, in human gastruloids model (Pauklin and Vallier, 2015; Liu et al., 2022). Essential for PS formation and extension, in chick and mouse (Lawson et al., 2001; Yanagawa et al., 2011; Pauklin and Vallier, 2015), *NODAL* expression in the PS succeeds the expression of the mesodermal marker gene, the T-box transcription factor *Brachyury* (*TBXT)* (Agius et al., 2000; Brennan et al., 2001; Takenaga et al., 2007). Moreover, two central BMP4 protein inhibitors, *Noggin* and *Chordin*, become upregulated at the anterior part of the PS where they are necessary for neural lineage commitment (Streit et al., 1998; Bachiller et al., 2000). Collectively, these findings suggest that while *BMP4* and its downstream pluripotency gene targets *NANOG* and *ID2*, are expressed and active during blastulation, they are down-regulated upon the onset of gastrulation, whereas genes which regulate PS formation and lineage-commitment, such as *NODAL*, *TBXT*, *Noggin* and *Chordin* are becoming upregulated. Nevertheless, it is unknown whether these molecular processes are affected by different storage conditions, which in turn, may impact the ability to successfully resume development.

This study aimed at investigating the initial molecular events of recovery in embryos exiting a prolonged storage at lower or higher temperature. Effects on blastoderm morphology and expression of key genes involved in pluripotency and differentiation were examined during the first hours of incubation at 37.8°C, referred hereinafter as the resumption phase.

## Animals, materials and methods

### Chick embryos

*Gallus gallus domesticus* freshly laid eggs from Ross (308) broiler breed were purchased from a commercial breeder. The flock age was 30–34 weeks. The eggs were laid in automatic nests, automatically collected, and delivered to the lab within 2.5–3 h from laying. The eggs were stored for 28 days in cooler incubator (VELP SCIENTIFICA, SN 265959, Italy) set at maintained temperature of either 12°C (28 days/12°C group) or 18°C (28days/18°C group) and in 70–80% relative humidity (RH). The temperature and RH were monitored using data logger (U-Sensor Plus, PN: 100475, United States). Freshly laid eggs were used as control, and were incubated upon delivery. Following storage, embryos were isolated as previously described (Pokhrel et al., 2018) either before incubation (fresh, 28 days/12°C, and 28 days/18°C groups), after 12 h of incubation at 37.8°C in 56% RH (fresh + 12 h, 28 days/12°C + 12 h, and 28 days/18°C + 12 h groups), or after 18 h of incubation (fresh +18 h, 28days/12°C + 18 h, and 28 days/18°C + 18 h groups). The incubator used in all experiments was Masalles model 65-I (Spain), equipped with data logger. Collectively, embryos isolated from the 9 treatment groups were collected for whole mount RNA *in situ* hybridization (WMISH) or for real time PCR.

For visualizing the morphological changes that occur during the resumption phase, 28 days/18°C stored eggs were windowed using fine scissors, and about 10 µl of 1% Fast Green dye was injected through the Vitelline membrane ventrally (underneath) to the embryo, using fine borosilicate glass tube, without rupturing the blastoderm. The blastoderm were visualized and pictured using dissecting microscope (Nikon, Model SMZ800, United States). For the time-course experiments, pictures were taken at time 0, and 3, 8, and 16 h of incubation later. During incubation periods, the windowed eggshells were sealed with parafilm.

### Whole mount *in situ* hybridization

Embryos were fixated in 4% paraformaldehyde (PFA) in phosphate buffered saline (PBS, Bioprep, Cat #PBSX10-1L, Israel) for overnight, as previously described (Weisinger et al., 2010, 2012; Kayam et al., 2013) and underwent WMISH analysis. At least 4 embryos per group were used for WMISH analysis. In brief, fixated embryos were washed three times with PBS and dehydrated in a series of 50%, 75%, 90% and 100% methanol (Biolab, Catalog No. 001368052100, Israel), each step for 20 min, at room temperature (RT). Embryos were stored in 100% methanol for at least 24 h at -20°C, and pooled to ensure the same hybridization conditions for all groups. The embryos were rehydrated in a series of methanol- 100%, 90%, 75%, 50% and washed twice with PBS- each step for 20 min at RT, and treated with deoxycholic acid (DOC) solution for 8 min for tissue permeabilization. DOC solution consists of 1% NP-40 (Merch, Cat No. 492016, Germany), 1% SDS (Hylabs, REF. BP716/500D, Israel), 0.5% DOC (Sigma, D-6750, United States), 0.5% Tris HCl (pH 8), 0.5% EDTA (pH 8), and 0.15 M NaCl. The embryos were washed twice with PBS containing 2% Tween20 (Amresco, CAS. 9,005–64–5, United States) (PBST), and fixated in 4% PFA for 20 min at 4°C. Embryos were washed twice with PBST (5 min each) and incubated at 70°C with pre-hybridization buffer for 2 h, which was replaced with pre-hybridization buffer containing 1–2 µg of dig-labeled RNA probe. RNA probes for *NANOG*, *ID2*, *TBXT*, *NODAL* and *Noggin* genes were prepared according to Roche Applied Science’s protocol (Roche Applied Science, Germany), and their forward and reverse sequences are provided in Table 1. Samples were hybridized with dig-labeled RNA probes for overnight at 70°C. Following hybridization, the samples were washed with solution X (25% formamide, Sigma-Aldrich, Cat No. F9037, United States; 0.2% SSC, pH 4.5; 0.1% SDS; in DDW) for 30 min, 4 times at 68°C. For detection reaction, the embryos were equilibrated in MABT (1M Maleic acid buffer pH 7.5, Roche, REF. 33813900, Germany; containing 2% Tween] at RT for 10 min for 4 times, which was followed by 2 h blocking (10% Normal goat serum (NGS), 1% BBR, Roche, REF. 11096176001, Germany, in MABT). Anti-DIG antibody (1:1,000 diluted in blocking solution, Roche, REF. 11093274910, Germany) was added, prior to an overnight incubation at 4°C. Washing of the Ab was done with MABT for 1 h, 3 times at RT, followed by equilibration with NTMT (0.1M NaCl; 0.1 M Tris-HCl, pH 9.5; 0.05 M MgCl_2_; 2% Tween, in DDW) twice for 10 min at RT. Coloration reaction was done with NBP/BCIP mix (BCIP, 1:1,000 diluted in NTMT, Promega, REF. 28526902, United States, and NBP 1:1,000 diluted in NTMT, Promega, REF. 28116602, United States). Color development in samples was periodically monitored and the reaction was stopped by washing the samples in NTMT (X1 time for 5 min) and TBST (X3 times, each for 10 min). Stained embryos were re-fixated in 4% PFA overnight at 4°C, washed in PBS for 3 times, and imaged for microscopy analysis (Nikon, Model SMZ800, United States).

**TABLE 1:** List of primers used for making the template for the RNA probes.

Genes	Forward primer	Reverse primer	Melting temperature (°C)(forward)	Melting temperature (°C)(reverse)	Product size	References
*NANOG* Full name: Nanog homeobox Gene ID: 100272166 https://www.ncbi.nlm.nih.gov/gene/100272166	CAG​CAG​CAG​ACC​TCT​CCT​TGA​C	CCA​AAG​AAG​CCC​TCA​TCC​TCC	63.8	64.4	595	(GEISHA, Darnell et al. (2007); Lavial et al. (2007)
*ID2* Full name: Inhibitor of DNA binding 2, HLH protein Gene ID: 395852 https://www.ncbi.nlm.nih.gov/gene/395852	CCTTTCGGAGCACAACCT	GAG​CGC​TTT​GCT​GTC​ACT​C	58.8	59.9	354	(Lorda-Diez et al., 2009)
*NODAL* Full name: Nodal growth differentiation factor Gene ID: 395205 https://www.ncbi.nlm.nih.gov/gene/395205	CGG​CTG​GGC​AGT​GTT​CAA​C	GCA​CCT​GGC​TGG​GCT​TGT​AGA​G	65.2	67.6	529	GEISHA (Darnell et al., 2007)
*TBXT* Full name: T-box transcription factor T Gene ID: 395782 https://www.ncbi.nlm.nih.gov/gene/395782	TTC​ATC​GCT​GTG​ACG​GCG​TA	AGG​GAG​GAC​CAA​TTG​TCA​TG	66.1	59.8	432	Stuhlmiller and García-Castro, (2012)

### cDNA preparation and real time-PCR analysis

Embryos were pooled at five biological repeats per group, and stored in RNA save solution (Biological industries, REF 018911A, Israel) for RNA extraction (as described in Pokhrel et al., 2022). Embryo tissues were homogenized in Bio-Tri RNA extraction solution (Bio-Lab, Catalog No. 009010233100, Israel) using a pestle (United States Scientific, REF 1415–5,390, United States) and a motor (Kimble, Catalog No. 7495400000, United States) and incubated for 5 min at RT. 1-Bromo-3-chloropropane (Sigma, B9673-200ML, United States) was added to the sample (at a ratio of 1:10 to the added volume of Bio-Tri RNA solution), vortexed vigorously, and the samples were incubated at RT for 10 min. Samples were then centrifuged (Eppendorf, SN 5409IM308416, Model 5427 R, Germany) at 4°C for 30 min at 14000 rpm. The aqueous upper layer was collected and equal volume of chilled iso-propanol (Gadot, CAS 67–63–0, Israel) was added and mixed. For mRNA precipitation the samples were stored overnight at -20°C, followed by centrifugation at 14000 rpm for 30 min at 4 °C. The RNA pellet was washed with 70% and 100% ethanol, dried at RT, resuspended in DEPC water (Biological Industries, 01-852-1A, Israel), and concentration was measured using Nanodrop (ThermoFisher Scientific, Model NanoDrop One C, United States). For cDNA synthesis, 1 µg of RNA was used according to the protocol of Promega cDNA synthesis kit (Promega, REF 017319, United States). For Real-time PCR 1 µl of cDNA was added to SYBR Green PCR Master Mix Kit (REF-4309155, Applied biosystems by Thermo Fisher Scientific, Inchinnan, United Kingdom) as previously described (Pokhrel et al., 2022), in the Applied biosystems Real-Time PCR Detection System (SN 2720011,007, Applied biosystems stepOnePlus Real-Time PCR System, Singapore). Samples were loaded in duplicates. Primers for the *NANOG*, *ID2*, *NODAL*, *TBXT* and *GAPDH* (as a normalizing gene) were designed for the qRT-PCR analysis according to sequence information from the NCBI database using Primer3 Input software (version V. 0.4.0) (Koressaar and Remm, 2007). Primer sequences are given in Table 2. The relative gene expression value was calculated using the 2^−ΔΔCT^ method. First, the obtained Ct value of each gene in each sample was normalized by the Ct value of *GAPDH* of the respective samples. The *GAPDH*-normalized expression value of the *NANOG*, *ID2*, *GATA4*, *NODAL* and *TBXT* genes in the different samples was again normalized with the *GAPDH*-normalized expression value of respective target genes of the fresh control samples. The obtained value was converted using the 2^−ΔΔCT^ formula. Thus, the obtained gene expression level is presented in fold change, which is relative to the control fresh embryos. Statistical analysis was done as described below.

**TABLE 2:** List of primers used for qRT-PCR.

Genes	Forward primer	Reverse primer	Melting temperature (°C)(forward)	Melting temperature (°C)(reverse)	Product size
*GAPDH* Full name: Glyceraldehyde-3-phosphate dehydrogenase Gene ID: 374193 https://www.ncbi.nlm.nih.gov/gene/374193	ACT​GTC​AAG​GCT​GAG​AAC​GG	ACC​TGC​ATC​TGC​CCA​TTT​GA	60.4	63.9	98
*NANOG* Full name: Nanog homeobox Gene ID: 100272166 https://www.ncbi.nlm.nih.gov/gene/100272166	CTC​TGG​GGC​TCA​CCT​ACA​AG	AGC​CCT​GGT​GAA​ATG​TAG​GG	59.9	61.3	167
*ID2* Full name: Inhibitor of DNA binding 2, HLH protein Gene ID: 395852 https://www.ncbi.nlm.nih.gov/gene/395852	CTG​ACC​ACG​CTC​AAC​ACA​G	TGC​TGT​CAC​TCG​CCA​TTA​GT	59	59.5	82
*NODAL* Full name: Nodal growth differentiation factor Gene ID: 395205 https://www.ncbi.nlm.nih.gov/gene/395205	GTC​CTG​CTG​CTC​GTC​TTC​TC	CCT​CTG​CCT​CTC​CTT​CCT​G	60.3	60.1	150
*TBXT* Full name: T-box transcription factor T Gene ID: 395782 https://www.ncbi.nlm.nih.gov/gene/395782	AAC​TCC​TCT​GCC​TGC​CTT​C	GTG​CTG​TTA​CTC​ACG​GAC​CA	59.5	59.8	155

### *In vivo* embryo treatment

Heparin acrylic beads (Sigma, Catalog No. H5263, United States) were soaked with 2 µl of recombinant Human Activin protein (Pepro Tech, Catalog No.1201450UG, Israel; 100 ng/µl; prepared by adding sterile 0.1% bovine serum albumin solution, Biological Industries, REF 030101B, Israel) or with PBS as control for 2 h at 4°C in ice. Embryos stored at 18°C for 28 days were incubated at 37.8°C for 12 h. A small window in the eggshell was opened through the air sac for *in vivo* transplantation of the Activin protein-soaked heparin beads. The beads were injected by a micropipette between the vitelline membrane and epiblast region. Following transplantation, the eggshell was sealed using leucoplast tape (BSN medical GmbH, REF 72668–02, Germany) and the embryos were reset for 6 h of incubation. Following incubation, the embryos were isolated, fixed overnight in 4% PFA and processed for RNA WMISH, as described above.

### Statistical analysis

Fold change expression levels of gene in different groups from at least three experiments were log2 transformed for normalization and analyzed by one-way ANOVA statistical tool (Tukey’s multiple comparison test). Data are presented as mean ± standard error of mean (SEM) that was calculated as the standard deviation divided by the square root of the count of samples. Statistical significance was determined at *p* < 0.05. Statistical calculations were done using Microsoft Excel and GraphPad Prism 6 software.

## Results

### Epiblast cells re-organize their morphology during the resumption phase

**FIGURE 1:**
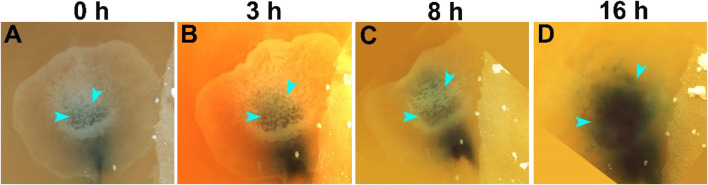
Morphological changes in embryos during the resumption phase. Embryos were stored for 28 days at 18°C (*n* = 22 embryos). The cellular changes during the resumption phase were demonstrated in a series of time-course images, at the beginning of incubation at 37.8°C, at time 0 **(A)**, and 3, 8, 16 h later (**B–D**, respectively). The dark color underneath the blastoderm is Fast Green dye, injected to highlight the contours of the embryos. While at the first hours of incubation after storage, the embryos are thicker with deep recesses in the epiblast (**A–C**, arrowheads), these recesses gradually disappear such that within 16 h of incubation, the recesses are no longer visible and the embryo becomes transparent (**D**, arrowheads).

We have previously described the morphological changes occurring following prolong storage, notably the thickening of the embryonic tissue and formation of deep recesses in the 28 days/18°C stored embryo (Pokhrel et al., 2018, 2022). Thus, to analyze the blastoderm morphological changes during the resumption phase of the 28 days/18°C group, embryos were incubated and in a time-course experiment, were imaged following 0, 3, 8, and 16 h (Figures 1A–D). In agreement with our previous findings, we found that following storage, the blastoderm displayed overt formed recesses in the dorsal epiblast (Figure 1A, blue arrowheads). However, within the first hours of incubation, the embryos gradually rearranged their cytoarchitecture, leading to the sequential disappearance of the deep recesses until they were no longer visible by 16 h of incubation (Figures 1B–D, blue arrowheads).

### Expression of the pluripotency-related genes during the resumption phase

**FIGURE 2:**
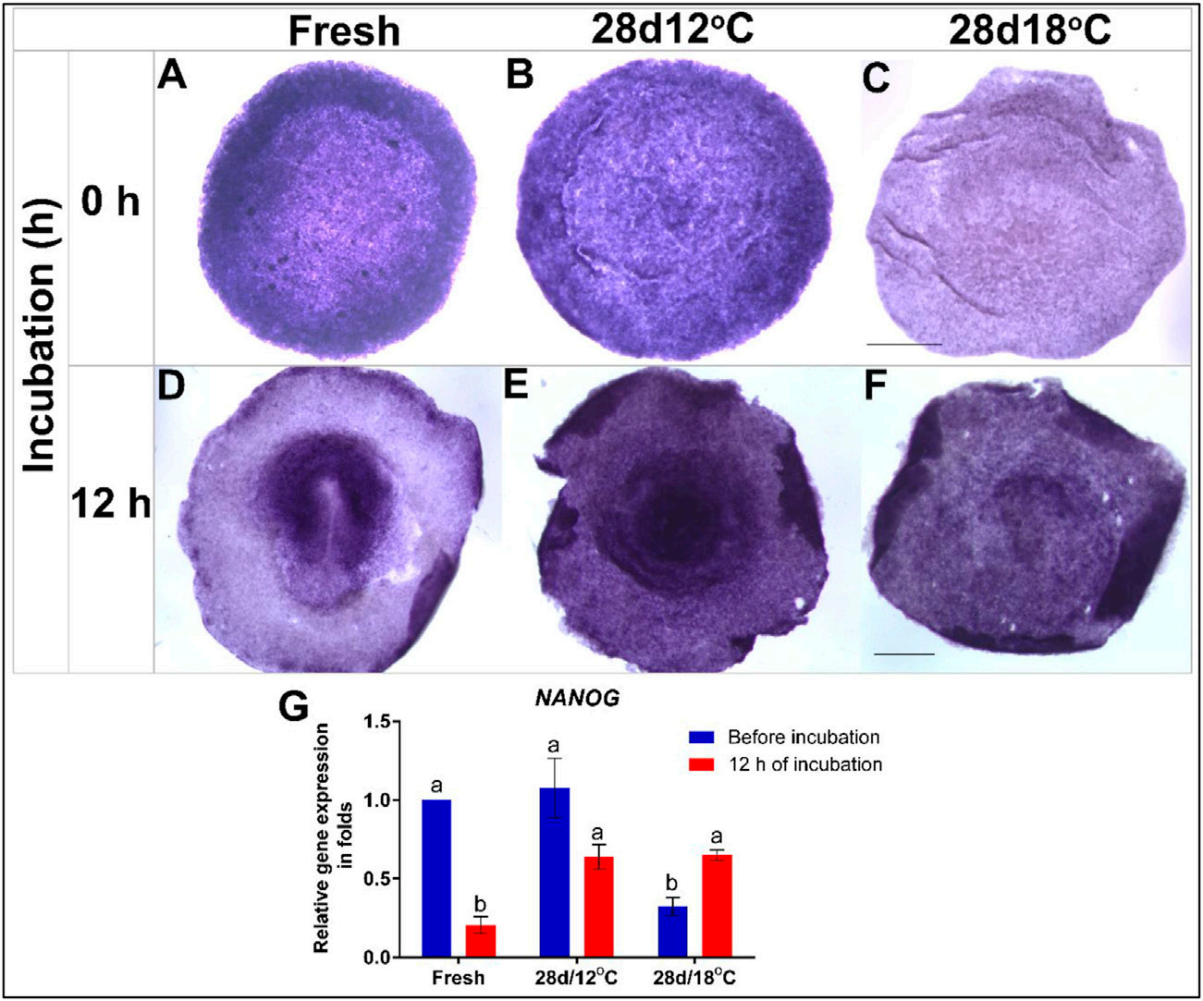
Expression of *NANOG* gene in embryos following storage, and following 12 h of incubation in the resumption phase. **(A–C)** WMISH analysis of *NANOG* expression in freshly-laid embryo (XI EG&K) or in embryos stored for 28 days at 12°C (XII EG&K), or 18°C (XIII EG&K). **(D–F)** WMISH analysis of *NANOG* expression following 12 h of incubation of the same embryonic groups. **(G)** Quantification of *NANOG* gene expression in 6 different embryo groups using RT-real-time PCR. *GAPDH* was used for normalization of *NANOG* gene expression. One-way ANOVA statistical analysis of 6 different embryo groups-different connecting letters denote that the expression of *NANOG* is significantly different between groups; F value = 16.51; Fresh, a vs. b: *p* < 0.0001; 28 days/18°C, a vs. b: *p* = 0.0357; Fresh (before incubation) vs. 28days/18°C (before incubation), a vs. b: *p* = 0.0008; 28 days/12°C (before incubation) vs. 28 days/18°C (before incubation), a vs. b: *p* = 0.0007; Fresh (after incubation) vs. 28 days/12°C (after incubation), a vs. b: *p* = 0.0023; Fresh (after incubation) vs. 28 days/18°C (after incubation), a vs. b: *p* = 0.0018). Bar = 1 mm.

**FIGURE 3:**
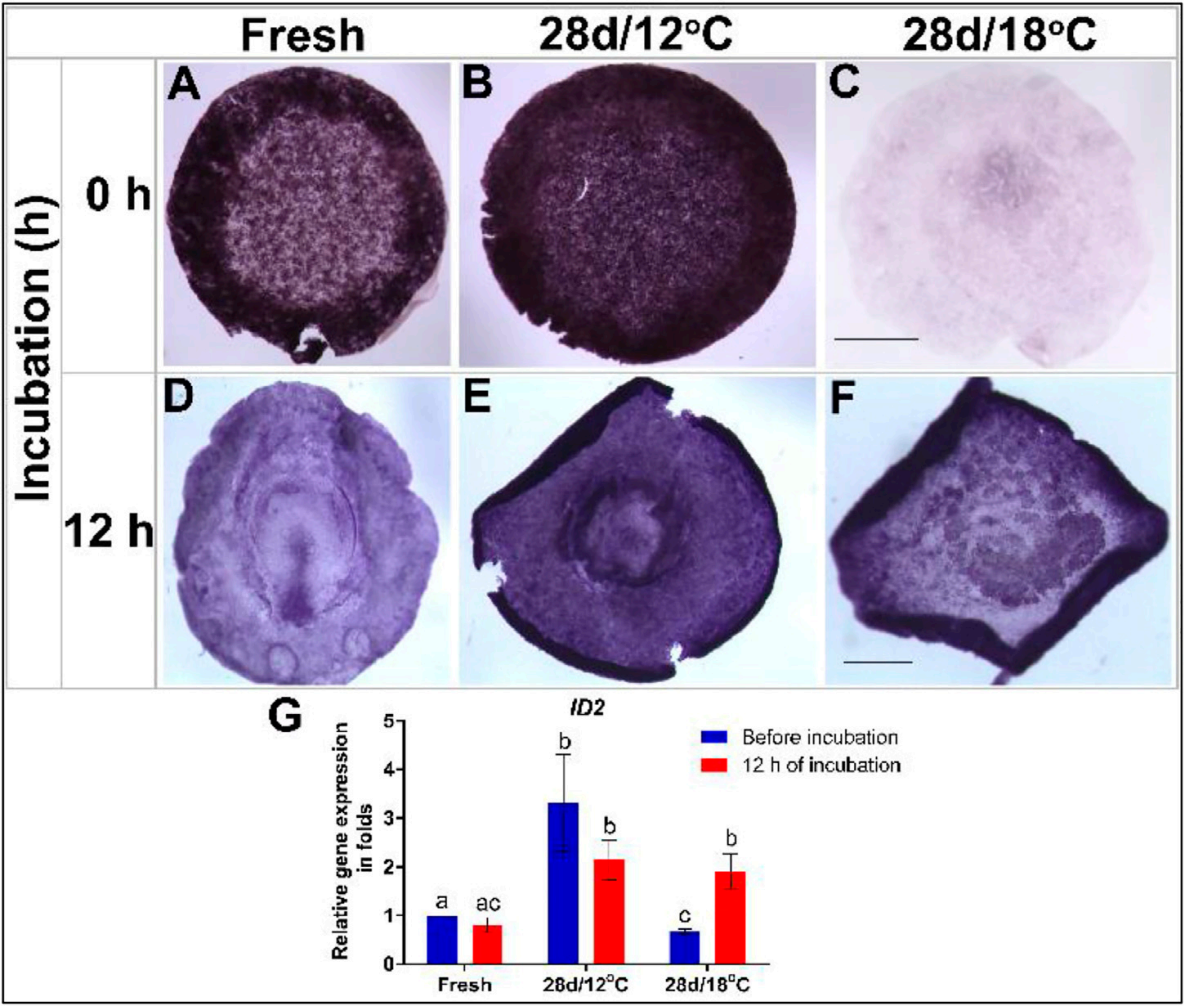
Expression of *ID2* gene in embryos following storage, and following 12 h of incubation in the resumption phase. **(A–C)** *ID2* gene expression in freshly laid embryo (X EG&K), embryo stored for 28 days at 12°C (XII EG&K), and embryo stored for 28 days at 18°C, respectively. **(D–F)** *ID2* gene expression during 12 h of resumption phase of embryos. **(G)** Quantification of *ID2* gene expression in 6 different embryo groups using real-time PCR. *GAPDH* was used for gene expression normalization and the determined *ID2* expression in the groups is represented as a fold of change relative to the fresh control. One-way ANOVA analysis of 6 different embryo groups-different connecting letters denote that the expression of *ID2* is significantly different between groups; F value = 11.83; Fresh (before incubation) vs. 28 days/12°C (before incubation), a vs. b: *p* = 0.0061; Fresh (before incubation) vs. 28 days/18°C (before incubation), a vs. c: *p* = 0.0079; 28 days/18°C, b vs. c: *p* = 0.0032; 28 days/12°C (before incubation) vs. 28 days/18°C (before incubation), b vs. c: *p* = 0.0002. Bar = 1 mm.

To investigate the changes in expression of pluripotency-related genes during the resumption phase, embryos of the 28 days/12°C and 28 days/18°C groups were analyzed before incubation or after 12 h of incubation, and compared to freshly-laid embryos which served as control (Figures 2, 3). The spatio-temporal expression pattern and levels of the examined genes were analyzed using WMISH and qRT-PCR. The expression of two genes, which are expressed during blastulation was analyzed–*NODAL* and *ID2*, are the results that are presented in Figures 2, 3, respectively.

#### NANOG

Broadly expressed in early embryonic cells, the pluripotency-associated homeobox transcription factor, *NANOG* is downregulated during gastrulation with the onset of mesodermal lineage commitment. Accordingly, *NANOG* expression in the control group was found throughout the epiblast during blastulation but as the embryos progress to gastrulation stage, and the PS has formed, *NANOG* expression decreased (Figures 2A,D,G). Similarity to control embryos, *NANOG* mRNA was also broadly expressed in the epiblast of the 28 days/12°C group following storage (Figures 2B,G), and following 12 h of incubation, *NANOG* expression level remained unchanged (Figures 2E,G). This is at variance from the control group which displayed lower *NANOG* expression at this stage (Figures 2D,G). This variation may possibly result from a developmental delay in the 28 days/12°C group following the prolong storage.

In contrast, *NANOG* expression was significantly reduced in the 28 days/18°C group at the end of storage (Figures 2C,G; fresh vs. 28 days/18°C, *p* = 0.0008; 28 days/12°C vs. 28 days/18°C, *p* = 0.0007). Interestingly, following 12 h of incubation of the 28 days/18°C group, *NANOG* expression got significantly upregulated (Figures 2F,G, *p* = 0.03), and became comparable with the 28 days/12°C groups (Figure 2G), suggesting that as far as *NANOG* expression, these embryos can regain their pluripotency state.

#### ID2

Essential for regulating the self-renewal characteristics and pluripotency of embryonic stem cells, the expression levels of the transcription factor *ID2* were similarly tested in embryos following 28 days of storage, before and after 12 h incubation and compared to freshly laid control embryos (Figure 3). Analysis of *ID2* expression pattern in non-incubated control embryos revealed its expression in the AO, AP, and polyingressing cells of the blastoderm (Figure 3A). Following incubation, *ID2* became restricted to the AP and PS (Figures 3D,G). A similar expression pattern of *ID2* was evident in different regions of the 28 days/12°C embryos prior to incubation (Figure 3B). However, *ID2* mRNA levels were significantly higher in this group compared to the control (Figures 3B,G, fresh vs. 28 days/12°C, *p* = 0.0061) and remained high after 12 h incubation (Figures 3E,G). At variance, a marked reduction in *ID2* expression was found throughout the blastoderm of embryos of the 28 days/18°C group prior to their incubation (Figures 3C,G; fresh vs. 28 days/18°C, *p* = 0.0079). Nevertheless, *ID2* expression was regained following 12 h of incubation (Figures 3F,G; 28 days/18°C before vs. after incubation, *p* = 0.0032) and became comparable to the 28 days/12°C group.

Collectively, these results demonstrate that expression of key pluripotent genes persist following storage at 12°C while gets down-regulated following storage at 18°C. However, at the resumption phase the affected embryos retain the ability to upregulate the expression of these genes, which may allow their normal initiation of development (Table 3).

**TABLE 3:** Summary results of expression of genes in embryos before and after incubation. Embryos were stored for 28 days at 18°C (28 days/18°C) or 12°C (28 days/18°C) and incubated at 37.8°C for 12 h for *NANOG* and *ID2*, and for 18 h for *NODAL* and *TBXT*. Freshly laid eggs, before and after incubation were used as controls. The embryos were isolated, fixed and stained by WMISH with *NANOG*, *ID2*, *NODAL*, and *Brachyury (TBXT)* probes. (−) indicates downregulated expression of the genes, whereas (+) indicates maintained or restored expression. The percentage of embryos in each groups and their count is given. Specific spatial expression in the Koller’s sickle (KS), Area Opaca (AO), Area pellucida (AP), or the Primitive streak (PS), is noted.

Genes	Incubation	Fresh (control)	28 days/12°C	28 days/18°C
*NANOG*	Before incubation	+ (100%, *n* = 6)	+ (100%, *n* = 6)	− (100%, n = 6)
	After incubation (12 h)	+ (100%, PS, *n* = 10)	+ (100%, AP, AO, *n* = 6)	+ (100%, AP, AO, n = 9)
*ID2*	Before incubation	+ (100%, *n* = 6)	+ (100%, AP, AO, *n* = 6)	− (100%, *n* = 6)
	After incubation (12 h)	+ (100%, PS, *n* = 13)	+ (91%, AP; 9%, PS, *n* = 11)	+ (100%, AP, AO, *n* = 7)
*NODAL*	Before incubation	+ (100%, KS, *n* = 9)	+ (100%, KS, *n* = 6)	− (92%, *n* = 13)
	After incubation (18 h)	+ (100%, PS, *n* = 7)	+ (100%, PS, *n* = 6)	− (75%, *n* = 4)
*TBXT (Brachyury)*	Before incubation	− (100%, *n* = 7)	− (100%, *n* = 6)	− (100%, *n* = 6)
	After incubation (18 h)	+ (100%, PS, *n* = 12)	+ (100%, PS, *n* = 6)	− (83.3%, *n* = 12)

### Early mesodermal inducing genes are down-regulated at the resumption phase following prolonged storage at 18°C

**FIGURE 4:**
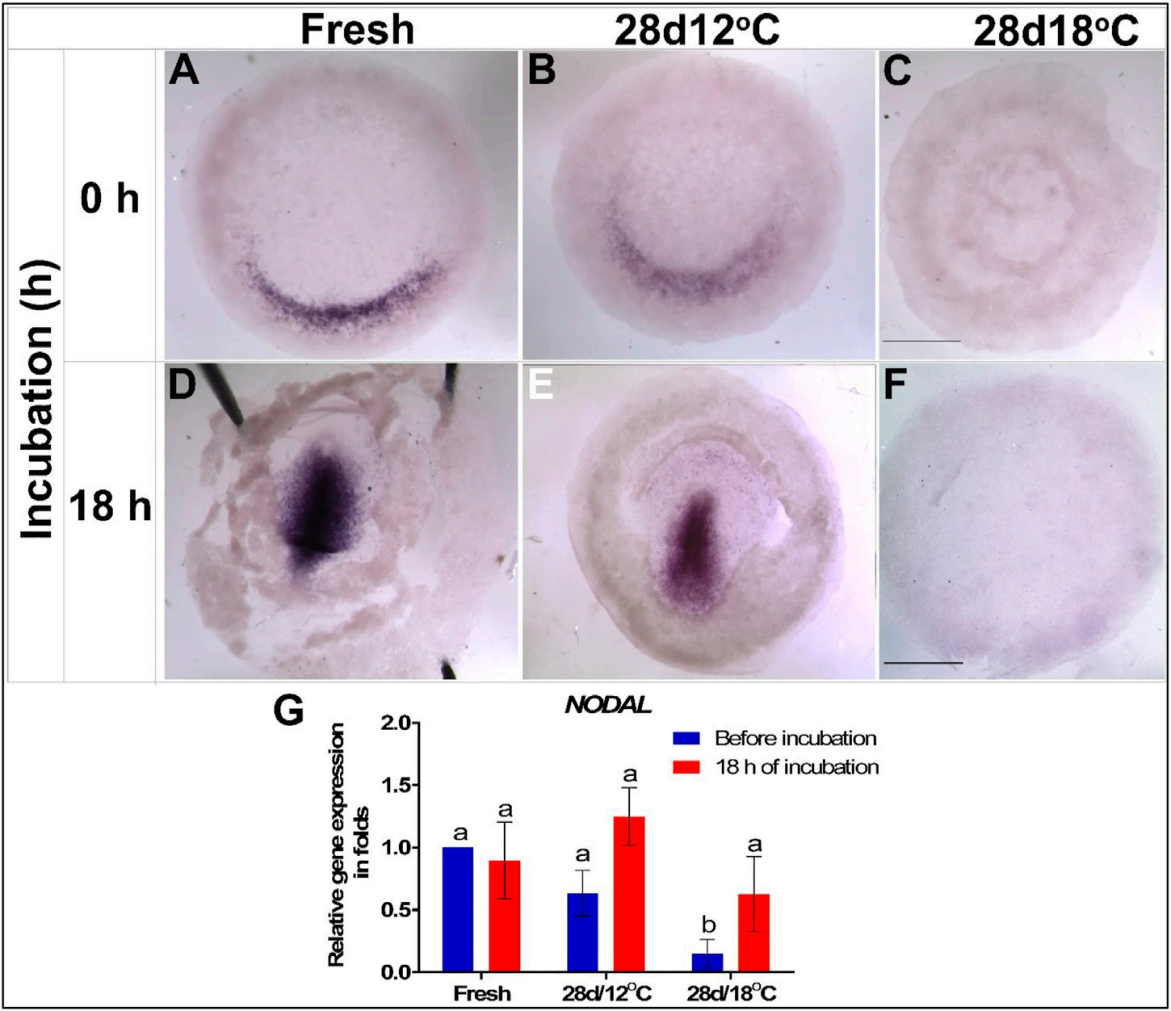
Expression of *NODAL* gene in embryos following storage, and following 18 h of incubation in the resumption phase. **(A–C)** *NODAL* gene expression in freshly laid (X EG&K), 28 days/12°C (XII EG&K), and 28 days/18°C (1 H&H) stored embryos, respectively. **(D–F)** Expression pattern of *NODAL* following 18 h of incubation, in freshly laid (3 H&H), 28 days/12°C (3 H&H), and 28 days/18°C (1 H&H) stored embryos, respectively. **(G)** Quantification of *NODAL* gene expression in 6 different embryo groups using real-time PCR. *GAPDH* was used for gene expression normalization and the determined *NODAL* expression in the groups is represented as a fold of change relative to the fresh control. One-way ANOVA analysis of 6 different embryo groups-different connecting letters denote that the expression of *NODAL* is significantly different between groups; F value = 7.913; Fresh (before incubation) vs. 28days/18°C (before incubation), a vs. b: *p* = 0.001; 28 days/12°C (before incubation) vs. 28 days/18°C (before incubation), a vs. b: *p* = 0.0098; 28 days/18°C (before incubation vs. after incubation), a vs. b: *p* = 0.025. Bar = 1 mm.

To investigate the effects of storage on the expression of markers that regulate gastrulation and specification of the mesoderm lineage, we studied the expression pattern of *NODAL* and its downstream effector *TBXT*. In the chicken embryo, *NODAL* expression begins at the posterior side of the embryo, in a crescent-shape structure in the Kollers’ sickle, and upon gastrulation, it gets upregulated within the PS (Chapman et al., 2007; Figures 4A,D, respectively). The expression pattern and levels of *NODAL* mRNA were tested in embryos of the 28 days/12°C and 28 days/18°C groups either before incubation or after 18 h of incubation, which correspond to gastrulation initiation (Figure 4). The results show that both, the non-stored control and the 28 days/12°C embryonic groups, express *NODAL* in the Kollers’ sickle in a similar manner, prior to incubation, and following 18 h of incubation *NODAL* is expressed along the PS (Figures 4A,B,D,E). In contrast, in the 28 days/18°C group, *NODAL* expression was absent before and after incubation (Figures 4C,F). qRT-PCR quantification of *NODAL* expression levels confirmed its downregulation in 28 days/18°C embryos compared with fresh (Figure 4G; *p* = 0.001) and 28 days/12°C embryos (*p* = 0.009). However, following 18 h of incubation, expression of *NODAL* mRNA was marginally restored in 28 days/18°C embryos (Figure 4G), but not to an extent that was noticeable by WMISH staining (Figure 4F). These result may suggest that 28 days/18°C embryos are impaired in their ability to induce *NODAL,* or are greatly delayed in development and therefore may induce *NODAL* expression at a later time point, during PS formation.

**FIGURE 5:**
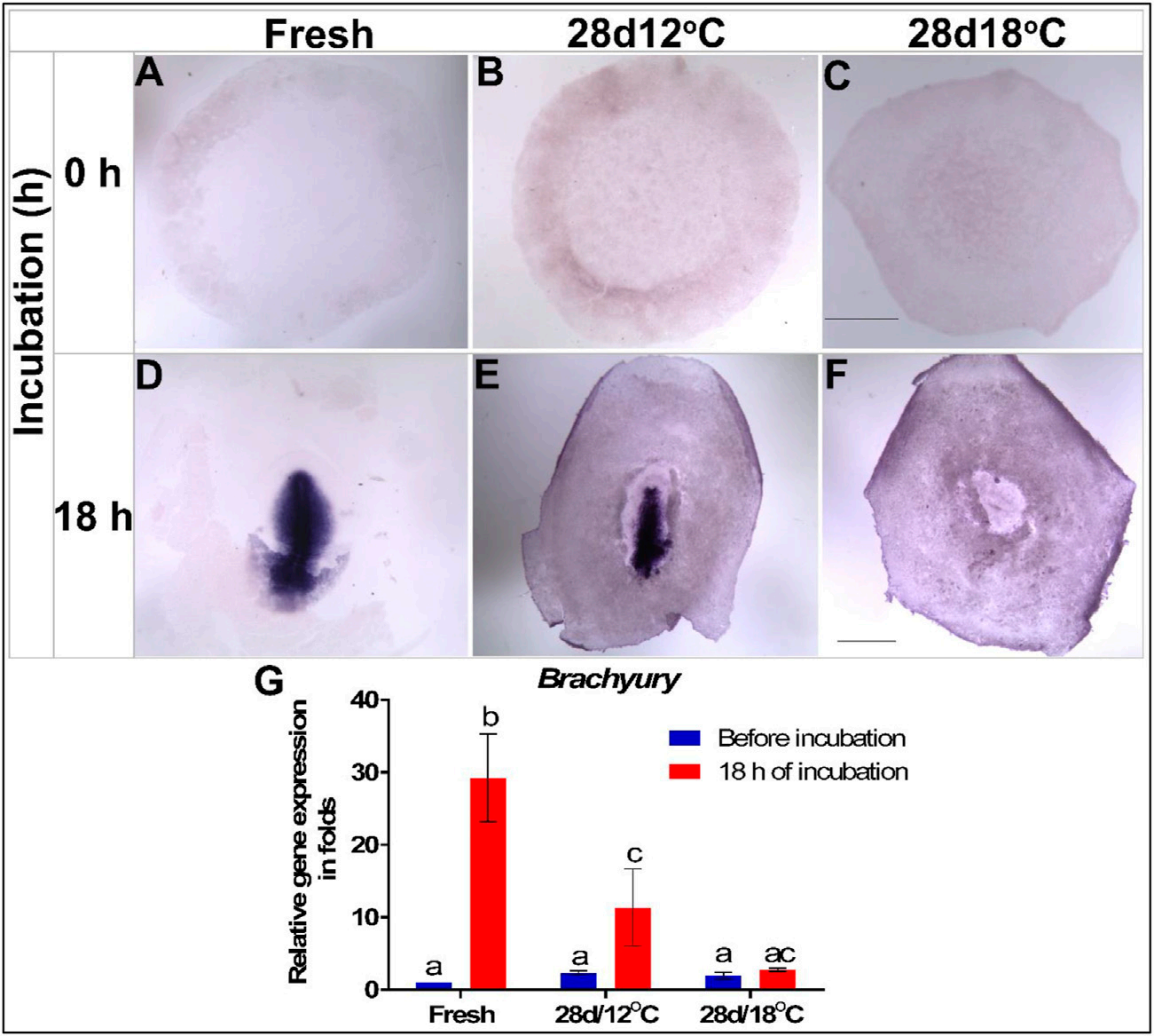
Expression of *Brachyury (TBXT)* gene in embryos following storage, and following 18 h of incubation in the resumption phase. **(A–C)** *TBXT* gene expression in freshly laid (X EG&K), 28 days/12°C (XI EG&K), and 28days/12°C (XIII EG&K) stored embryos, respectively. **(D–F)** Expression pattern of *TBXT* following 18 h of incubation, in freshly laid (3 H&H), 28 days/12°C (3 + H&H), and 28 days/12°C (1 H&H) stored embryos, respectively. **(G)** Quantification of *TBXT* gene expression in 6 different embryo groups using real-time PCR. *GAPDH* was used for gene expression normalization and the *TBXT* expression in the groups is represented as a fold of change relative to the fresh control. One-way ANOVA analysis of 6 different embryo groups; F value = 23.64; different connecting letters denote that the expression of *TBXT* is significantly different between groups; Fresh, a vs. b: *p* < 0.0001; 28 days/12°C, a vs. c: *p* = 0.007. Bar = 1 mm.

To further explore this possibility, the mRNA expression pattern and levels of the downstream effector of *NODAL*–*TBXT* (Pauklin and Vallier, 2015), was similarly tested (Figures 5A–G). The first expression of the *TBXT* in control embryos was noticeable following 18 h of incubation in the PS of the early gastrulating embryo (Figures 5D,E). As in the case of *NODAL*, *TBXT* expression in the 28 days/12°C embryos was comparable to that of the control group before and after incubation (Figures 5B,E). However, in accordance with the low expression of *NODAL* in the 28days/18°C embryos during the resumption phase (Figures 4F,G), WMISH showed that *TBXT* expression was also missing in this group (Figures 5C,F). Quantification of *TBXT* gene expression showed that the fresh and 28 days/12°C embryos significantly up regulated *TBXT* expression after 18 h of incubation (Figure 5G; fresh before vs. after incubation, *p* < 0.0001; 28 days/12°C before vs. after incubation, *p* = 0.007), whereas in 28 days/18°C embryos, the expression level of *TBXT* before and after incubation remained lower (Figure 5G; 28 days/18°C, *p* = 0.9348; Table 3).

### Activin ectopic administration rescues *NODAL* and *TBXT* expression

**FIGURE 6:**
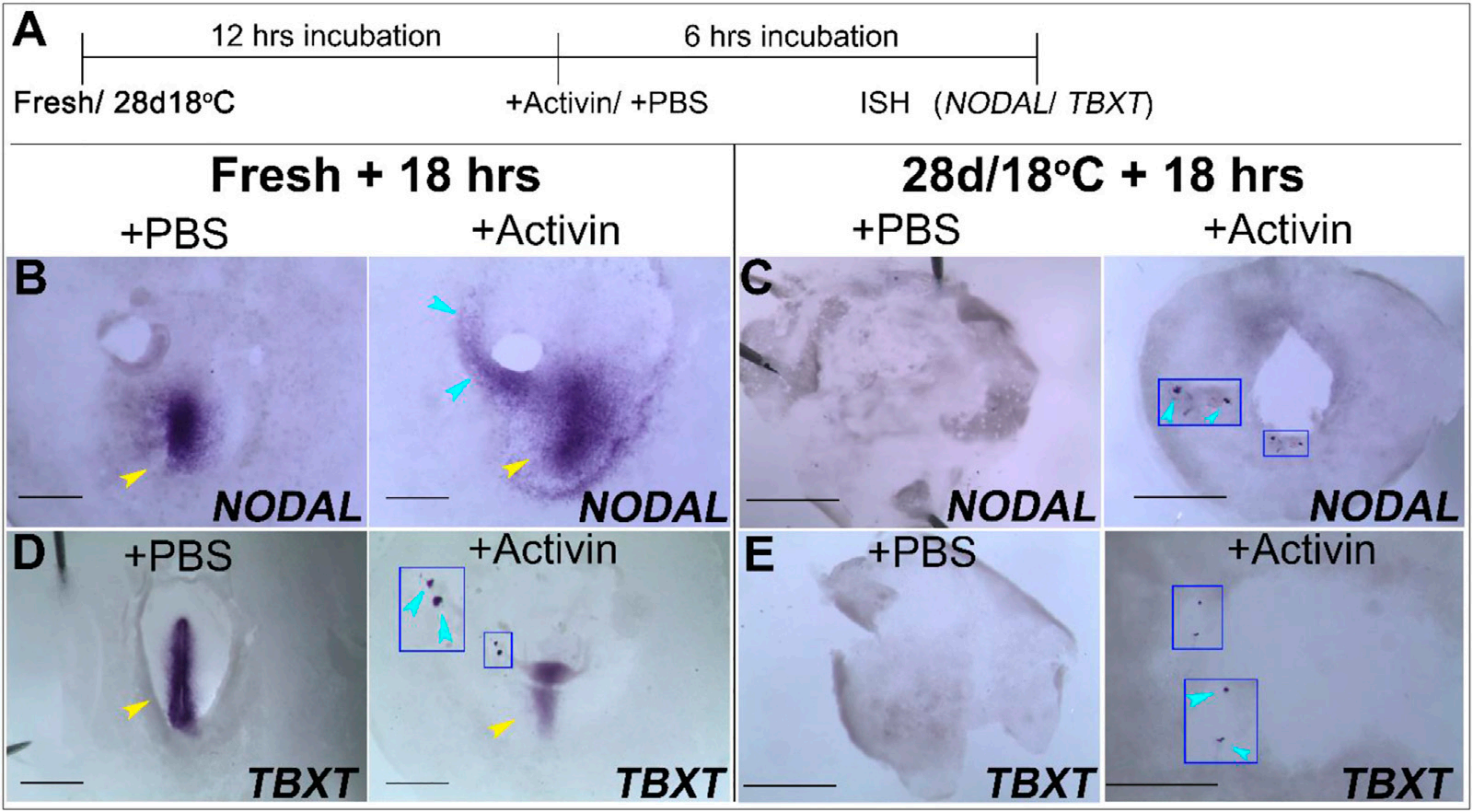
Ectopic application Activin induce the expression of *NODAL* and *TBXT* in fresh and 28 days/18°C embryonic groups. **(A)** Schematic illustration of the experimental design. Freshly laid embryos or 28 days/18°C stored embryos were incubated for 12 h at 37.8°C and then treated with PBS- or Activin-soaked bead for 6 h. Embryos were analyzed for *NODAL* and *TBXT* expression by WMISH. **(B**,**D)** WMISH analysis of *NODAL* and *TBXT* expression in fresh embryos following treatment with PBS or Activin protein. While the mock control fresh embryo treated with PBS-soaked beads expressed *NODAL* and *TBXT* gene in PS region only (yellow arrowhead, *n* = 4/4; 4/4, respectively), transplanting Activin-soaked beads in embryos, induced *NODAL* and *TBXT* expression near the transplanted beads (blue arrowhead, *n* = 4/4; 3/4, respectively, inset in **D** shows induced *TBXT* gene expression in high magnification view) as well as its normal expression in PS region (yellow arrowhead). (**C**,**E**) WMISH analysis of *NODAL* and *TBXT* expression in 28 days/18°C embryos following treatment with PBS or Activin protein. While in control embryos no *NODAL* and *TBXT* expression was induced (*n* = 4/4; 3/3, respectively), in Activin-treated embryos *NODAL* and *TBXT* expression were evident in the vicinity of the implanted beads (**C**,**E**, *n* = 5/5; 5/7, respectively; insets in **C**,**E** and blue arrowheads show induced *NODAL* and *TBXT* gene expression in high magnification view). Bar = 1 mm.

The observation that the PS markers *NODAL* and *TBXT* are not upregulated in the 28 days/18°C stored embryos following 18 h of incubation at 37.8°C (Figures 4, 5; Table 3), correlates with their poor embryonic viability and hatchability (Pokhrel et al., 2018). In order to determine whether these embryos can restore the expression of these genes and enter gastrulation, a rescue experiment was designed using ectopic administration of Activin that induces *NODAL* expression. Embryos that were stored at 28 days/18°C and incubated for 12 h were implanted with acrylic heparin beads (Korn and Cramer, 2007; Mohammed and Sweetman, 2016) soaked with PBS or recombinant Activin, and re-incubated for additional 6 h, to allow them to progress into gastrulation (Figure 6A). Freshly laid embryos were also incubated with PBS or Activin-soaked beads for the same duration to serve as mock controls and positive controls, respectively (Figures 6A,B,D). Embryos of all groups were then examined for *NODAL* and *TBXT* expression by WMISH (Figure 6A).

Our results show that exogenous application of Activin in control freshly laid embryos was able to induce ectopic expression of *NODAL* and *TBXT* in the PS region and near the grafted beads, in contrast to the PBS-added embryos (Figures 6B,D). Similarly, addition of Activin to the 28 days/18°C embryos resulted in upregulation of *NODAL* and *TBXT* in the vicinity of the transplanted beads, as opposed to their absence in the PBS- mock group (Figures 6C,E; see also Figures 4F, 5F). These results suggest that while prolonged storage at 18°C prevents the upregulation of gastrulation-related genes, the ability to enter gastrulation persists in the embryos upon receiving the instructive exogenous signals.

## Discussion

The ability to suspend embryogenesis during blastulation allows the avian embryo to withstand prolong developmental arrest when kept at low temperatures, and resume normal development at appropriate incubation conditions thereafter (Brake et al., 1997; Reijrink et al., 2008). This arrest occurs at a critical developmental stage of the transition from blastulation to gastrulation, when the foundations for all the embryonic germ layers are set. The effects of prolong developmental arrest was previously studied in turkey breeding eggs, which were stored for up to 27 days, and the developmental stage following 8 days of incubation was evaluated. Embryos that were stored for 5 days or less were significantly more advanced developmentally than the embryos from eggs stored for longer (Bakst et al., 2016) highlighting the potential deleterious effects of storage conditions, and the importance of understanding the phenomenon for better hatcheries managerial decisions. We have previously shown the deleterious effects of prolonged storage at 18°C compared with 12°C, on embryonic survivability and hatchability. At 18°C, only about 17% of embryos which were stored at 18°C for 28 days were able to survive and hatch (Pokhrel et al., 2018). The surviving hatchlings highlight the robustness of some embryos to endure severe environmental conditions, however, the mechanisms which promote these abilities are poorly understood. This study investigated the cellular and molecular characteristics of embryos exiting storage and categorized the transition period from storage to exiting storage as the resumption phase. Broadly, the storage period can be divided into 2 phases, the first—the initiation phase, which begins following laying and up to 7 days, during which embryonic survival improves (Brake et al., 1997), and the second—the extension phase, which extends beyond 7 days of storage and may deteriorate survivability depending on environmental conditions. Notably, as the extension phase is highly sensitive to the temperature (Pokhrel et al., 2018), a successful resumption phase, which marks the transition between storage and the first hours of incubation, is critical for allowing successful developmental progression and hatching.

The effects of environmental conditions during storage were investigated for many years (Bakst and Akuffo, 2002), emphasizing that storage temperature and duration are the critical factors for later embryonic survivability (Reis et al., 1997; Gomez-de-Travecedo et al., 2014). These factors were shown to affect physical and biological properties of embryos within the eggs, and hatchability and chick quality post hatch (Brake et al., 1997; Reijrink et al., 2008; Hamidu et al., 2011; Özlü et al., 2021), indicating that changes in these properties perturb the ability to resume normal development. In agreement with these studies, our results suggest that embryos undergoing storage at lower or higher temperatures, evoke differently biological processes, which promote their ability to successfully resume development during the resumption phase. These include an adaptive cytoarchitectural changes and recovery of expression of genes associated with pluripotency, *NANOG* and *ID2* (Ying et al., 2003; Lavial et al., 2007; Pan and Thomson, 2007; Luo et al., 2012; Yu et al., 2020). Notably, BMP4 signaling was previously found to induce expression of *NANOG* and *ID2* in mouse embryonic stem cells and in chick blastoderm cells (Ying et al., 2003; Lavial et al., 2007; Pan and Thomson, 2007; Luo et al., 2012; Yu et al., 2020; Pokhrel et al., 2021a), raising the possibility that while BMP4 signaling is preserved during storage at 12°C, it is not maintained at 18°C, but can be reactivated after incubation of these embryos, leading to upregulation of *NANOG* and *ID2*. Notably, the expression of *NANOG* was broadly distributed in the AP region of the post-storage embryos, as opposed to previous studies which showed diminished expression of this gene, in the PS region (Lavial et al., 2007), suggesting a possible developmental delay in embryos following longer storage period at both storage temperatures. For instance, the developmental delay in the 28 days/18°C groups may be due to the recovery time required for embryos to rearrange the cytoarchitecture and restore the lost gene expression. However, activation of the BMP4 pathway as a recovery process might have accounted delay in formation of PS because downregulation of the BMP4 pathway is required for initiation of PS formation (Streit et al., 1998; Bertocchini and Stern, 2008; Vasiev et al., 2010; Supplementary Figure S1). Likewise, the delayed PS formation in the 28 days/12°C group could be due to a delay in the reactivation mechanisms associated for transition to gastrulation stages, perhaps due to the prolonged activation of the BMP4 pathway during storage. This delay in the initial developmental events during the resumption phase of prolong stored embryos at 12°C and 18°C may be responsible for the spread in the hatching time window from long-stored eggs, as observed previously (Tona et al., 2003). The changes in RNA levels presented here require further validation in future studies at the protein level to fully confirm the involvement of the examined genes in the ability to resume development. However, the lack of available chicken-specific antibodies for these transcription factors prevent these assessments.

Interestingly, in contrast to the ability to restore the expression of *NANOG* and *ID2* after 12 h of resumption phase of the 28 days/18°C group, this did not hold for the initiation of PS formation and the restoration of mesoderm specification genes *NODAL* and *TBXT* even after 18 h of incubation. This finding varied from the ability of 28 days/12°C embryos to induce *NODAL* and *TBXT* expression in the PS region after the resumption phases, consistently with previous studies (Chapman et al., 2002; Mikawa et al., 2004). Notably, after incubation of some 28 days/12°C embryos, *NODAL* gene expression was restricted to the truncated region of PS, further highlighting that the onset of gastrulation is somewhat delayed in these embryos. Moreover, the finding that during the resumption phase of 28 days/18°C embryos, expression of some, but not all, examined genes can be restored suggests that the plasticity of these embryos to restore gene expression is limited to a few genes. Moreover, since the timing of *NODAL* gene expression in zebrafish embryos at stages from mid-blastula to mid-gastrula (van Boxtel et al., 2015) regulates the timing of mesendodermal induction and lineage commitment at later stages (Dougan et al., 2003; Weng and Stemple 2003; Fleming et al., 2013), the inability to restore these gastrulation-related signaling pathways following prolonged storage at 18°C may explain the broad embryonic mortality and the reduced hatching rate in these conditions (Pokhrel et al., 2018; 2021b). Finally, investigating the cellular and molecular dynamics of embryos during and after storage also revealed that embryos are viable during prolong storage at 18°C and restore development after incubation, however, they lack the molecular processes essential for differentiation and fail to develop upon incubation.

Notably, our findings regarding the association between storage temperature and the ability to induce gastrulation-related genes during the resumption phase is in agreement with other studies showing that modification of storage conditions can improve the successful resumption into development (Kgwatalala et al., 2013; Brady et al., 2022).

Hence, our data further highlight the interaction between developmental stage and the temperature during storage, and suggests that reactivation mechanisms are maintained during storage at low or high temperature to upregulate signaling pathways associated with pluripotency to facilitate successful resumption into development. Moreover, even the inability of the 18°C stored embryos to undergo gastrulation transition could be rescued by adding exogenous signals, further indicating that thermal manipulations can revert the negative effects of storage at high temperature on embryonic development.

The current study focused on previously characterizing genes which are known to be involved in maintaining pluripotency or differentiation in prolonged stored blastoderms. Other mechanisms are clearly involved in maintaining embryonic survivability. Recent studies have begun to uncover the molecular mechanisms that participate during storage by transcriptome profiling (Pokhrel et al., 2021a, 2022; Brady et al., 2022). These studies reported and highlighted the roles of cell-cycle, cellular death, metabolism, ubiquitination, and cytoskeleton-related mechanisms, which partake in embryonic survivability and enable embryos to successfully resume development. Along with these molecular mechanisms, the embryonic surrounding environment, namely the yolk and albumen properties, is known to be affected by storage condition, and affect embryonic survival. For instance longer periods of storage resulted in lower albumen weight and height, together with higher albumen pH, as opposed to freshly laid eggs (Scott and Silversidest, 2000; Silversides and Budgell, 2004). Identification of differences in additional molecular and physiological processes which relate to different storage conditions could be beneficial for the poultry industry to calibrate storage conditions and improve the levels of appropriate embryonic development and hatching.

## Conclusion

Environment temperature plays a crucial role in avian embryonic development during storage and affects the ability of embryos to successfully resume development when incubated. Particularly, embryos that enter storage at lower temperatures, have higher chances to resume normal development than embryos which storage at higher temperatures. The overall decrease in metabolic activity at lower temperature may contribute to embryonic survivability, however, even at low storage temperature the embryo induces survival mechanisms and signaling pathways, which protect its morphology, cells viability, and pluripotency state, thereby allowing it to successfully resume normal development. Nevertheless, the molecular regulation that enables stored embryos at higher or lower temperatures, to resume development, is mostly unknown. In this study, we investigated how prolong storage at 12°C vs. 18°C affect the embryonic ability to resume development by examining their cellular and molecular dynamics following exit from storage. Our results show that embryos maintain the ability to express genes associated with pluripotency and differentiation during storage. These pathways are essential for the transition from blastulation to gastrulation during the successive development event at the time of exiting storage. Notably, embryos stored at lower or higher temperature display differences in expression of gastrulation-related genes, which could explain the divergence in resumption of development and thus the differences in embryonic survival at hatching following storage at different temperatures. Collectively, this study suggests for the first time a temperature-dependent mechanism that enables the transition from blastulation-to-gastrulation stages in order to promote successful development following prolonged storage.

## Data Availability

The original contributions presented in the study are included in the article/Supplementary Materials, further inquiries can be directed to the corresponding authors.
