# Supplementary material for: Storage temperature dictates the ability of chicken embryos to successfully resume development by regulating expression of blastulation and gastrulation genes
Source: Front Physiol. 2022 Dec 16;13:960061. doi: 10.3389/fphys.2022.960061 (PMC9800875; doi:10.3389/fphys.2022.960061)
Supplement: Supplementary file 1 [file Image1.pdf]

Storage Temperature Dictates the Ability of Chicken Embryos to Successfully Resume Development by Regulating Expression of Blastulation and Gastrulation Genes

Supplementary Figures

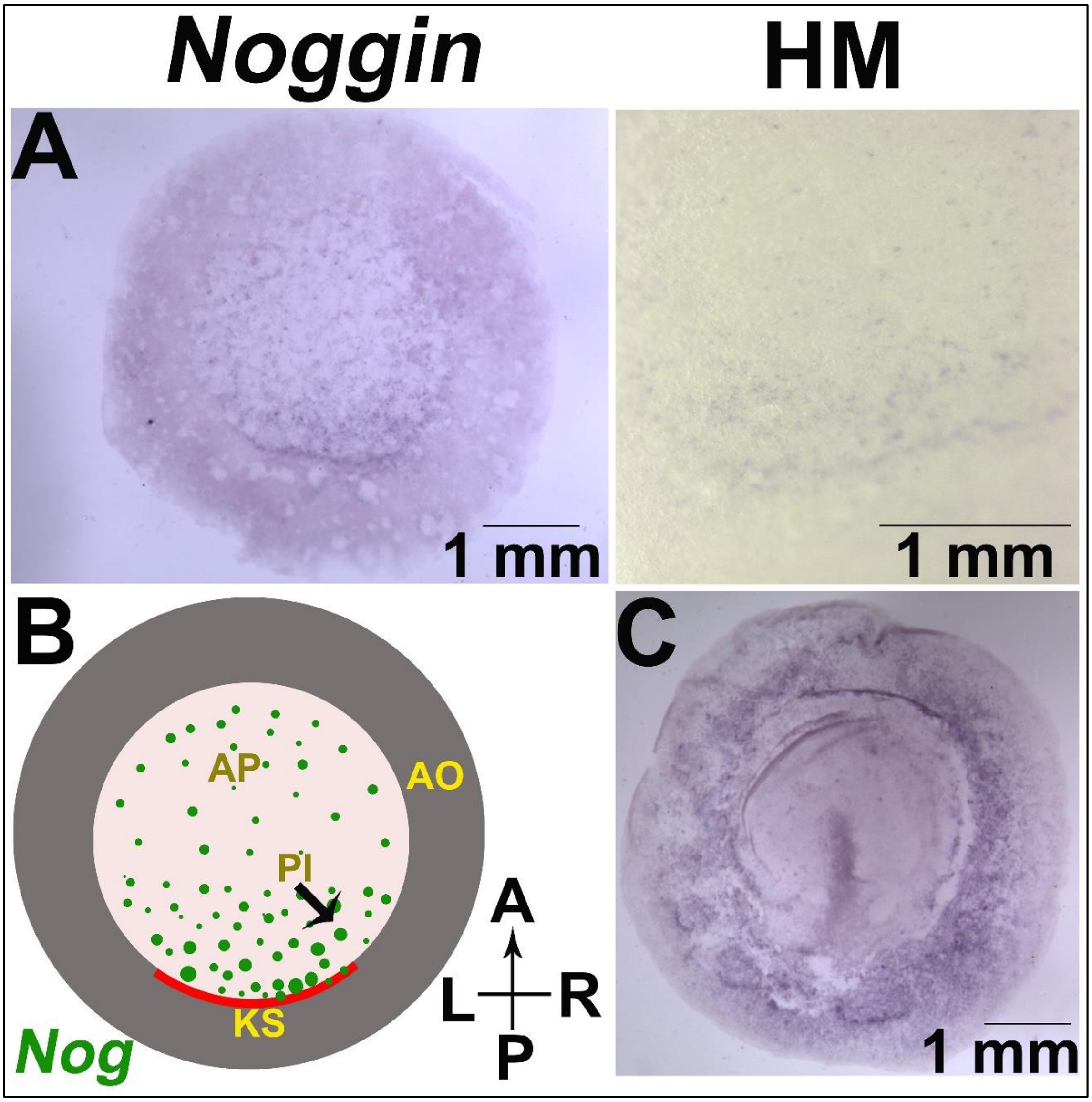

Figure. S1. Noggin expression in blastulating and early gastrulating embryos (HH2). **(A)** WMISH showing *Noggin* expression in polyingression cells and in Koller's sickle region of blastulation stage embryos. Right panel shows the high magnification (HM) image of panel A. **(B)** Schematic representation of Noggin expression in blastulation stage embryos. **(C)** WMISH showing *Noggin* expression in HH2 stage embryos. Noggin is expressed in primitive streak region of embryos. HM- High magnification; AP- Area pellucida; AO- Area opaca; KS- Koller's sickle; PI-Polyingressing cells; Arrow shows orientation of embryo (A-Anterior, P-Posterior, L- Left, R- Right). Bar size =1mm.
